# Management of the oral hemangiomas in infants and children: Scoping review

**DOI:** 10.4317/medoral.23329

**Published:** 2020-01-22

**Authors:** Alejandra Barrón-Peña, Martha A. Martínez-Borras, Oscar Benítez-Cárdenas, Amaury Pozos-Guillén, Arturo Garrocho-Rangel

**Affiliations:** 1 DDS, Especialidad en Estomatología Pediátrica, Facultad de Estomatología, Universidad Autónoma de San Luis Potosí, San Luis Potosí, S.L.P., México; 2 DDS, Departamento de Cirugía Oral y Maxilofacial, Facultad de Estomatología, Universidad Autónoma de San Luis Potosí, San Luis Potosí, S.L.P., México; 3 DDS, MSc, PhD, Especialidad en Estomatología Pediátrica, Facultad de Estomatología, Universidad Autónoma de San Luis Potosí, San Luis Potosí, S.L.P., México

## Abstract

**Background:**

Pediatric oral hemangiomas are benign vascular tumors that can be seen from birth, particularly in females. Hemangiomas are most frequent located in the lips and usually regress spontaneously, thus they do not require any type of treatment in most cases. The present scoping review pretended to synthesize the most relevant and currently available information from the international dental literature published in the last 25 years, regarding the management of pediatric oral hemangiomas.

**Material and Methods:**

An exhaustive literature search was performed in four electronic databases (PubMed, Embase, Google Scholar, and Cochrane). Initially, 241 related titles and abstracts were found. After the duplication removal, screening, and assessment processes, 37 records were included for full-text reading. Finally, 20 articles in the English language were included in the scoping review for data extraction and assessment.

**Results:**

We identified and subsequently discussed three fundamental issues associated to the management of pediatric oral hemangiomas: (i) clinical characteristics, differential diagnosis, and histopathological findings; (ii) evolution and complications; and (iii) current available treatment modalities.

**Conclusions:**

Although these like-tumor lesions are uncommon, pediatric dentistry practitioners must be familiar with the inherent clinical characteristics, diagnosis approaches, and currently available treatment options. Nowadays, surgical removal and non-invasive medical/pharmacologic therapies are the best management modalities for pediatric oral hemangiomas.

** Key words:**Vascular tumors, hemangioma, oral management, children, scoping review.

## Introduction

According to the American Academy of Pediatric Dentistry (AAPD), there has been described numerous lesions, masses, or tumor-like conditions of soft and hard tissues, belonging to the oral and maxillofacial regions of children and adolescents; the majority of these lesions are mucosal conditions (1). Intra-oral mucosal lesions in American children between 2 and 17 years old have an incidence greater than 9%, being the vascular tumors the most common benign lesions (2). Oral vascular tumors encompass a wide spectrum of congenital and neonatal anomalies whose predominant components are vascular structures (3). The single term hemangioma (HEM) has been used in the medical/dental literature to describe the localized benign vascular tumor of infancy/childhood of mesenchymal origin (4,5). However, this term has been also employed for a wide range of mucosal/skin vascular pathologies, including “strawberry”, “port-wine”, and “salmon patch” (3,6). This varied terminology has led to confusion among clinicians in the diagnosis and treatment of these tumors (7). Likewise, it has extensively discussed if hemangiomas should be considered as neoplasms, hamartomas, or vascular malformations (4).

The vascular lesions of childhood are classified into two categories: hemangiomas (proliferating or involuting) and vascular malformations (8). Later, this classification was modified by the International Society for the Study of Vascular Anomalies in 1996 (9,10), in which vascular lesions were subdivided into (i) tumors (infantile and congenital hemangioma, pyogenic granuloma, and other rare entities) and (ii) vascular malformations.

HEM is the most common benign tumor of the blood vessels in infants and children; 80% of these tumors are present as isolated entities (11). This lesion is found occasionally in the mouth -along with lymphangiomas account for up to 30% of oral cavity tumors in the pediatric population- (2). It is observed more frequently in the lips, oral mucosa, cheek, tongue, palatal mucosa, salivary glands, and mandibular bone; and in the skin of the head, face, and neck (12). It has an incidence between 3% and 10% by the age of one year (13). The condition is more common in premature low-birth-weight infants (< than 1000 g), decreasing gestational age, in white female children, and in twins (9); other related risk factors are multiple gestation pregnancy, gestational hypertension, placenta previa, preeclampsia, chronic villus sampling, and antenatal vaginal bleeding (4,14). It occurs usually during the neonatal period (6,15). The tumor exhibits rapid growth and expansion with endothelial cell proliferation during the first five or six months of life, followed by a gradual self-involution to near-complete resolution (4); approximately 50-80% of all HEM disappear by 5 years of age (2). When needed, treatment can be surgical and/or pharmacological (16).

The aim of the present scoping literature review is to explore, describe, and discuss the most current evidence about the management of HEMs in the oral cavity of infants and children.

Material and Methods 

For the present scoping review, we judiciously followed the guidelines of Preferred Reporting Items for Systematic Reviews or Meta-Analyses Extension for Scoping Reviews (PRISMA-ScR) (17), and the recommendations of Arksey and O’Malley (18), and Levac *et al*. (19) methodologies. The next practice-orientated research question was structured: What is the best current evidence about the management of oral hemangiomas in infants and children?

A comprehensive literature search was carried between January to May 2019, using four electronic databases: PubMed, Embase, Google Scholar, and the Cochrane Library. The included articles were limited to English and Spanish languages only, published during the last 25 years, with the following methodological designs: narrative/systematic reviews randomized clinical controlled trials (RCCTs), cohort and case and control studies, and clinical case reports; focused on clinical/histological characteristic, diagnosis and the different treatment modalities, and other useful information about oral and peri-oral localized hemangiomas in infants and children (since birth up to 12 years old). Guest editorials, clinical opinions, abstracts, gray literature, conference reviews, and meeting highlights were excluded. The principal keywords and MeSH terms employed, alone or in combination with Boolean operators, for the different searches were as follows: “oral vascular tumors OR lesions”, “oral hemangioma”, “infantile hemangioma”, “congenital hemangioma”, AND “children OR pediatric patients”. This search strategy was adapted for use in every database.

Related titles and abstracts were identified and assessed by two pre-calibrated independent authors (ABP and OBC) for eligibility, after eliminating duplicated articles. The studies that appeared to meet the inclusion criteria were retrieved in their full-text version and evaluated. A manual search for additional relevant titles was also carried out in the bibliographic reference list reported by each included article. After that, useful data (first author’s name, publication year, country, research design, sample size (in cases of RCCTs, cohort, or case-control studies), and author’s main reported outcomes, findings, and recommendations) were extracted from each study, using a pre-piloted specific form; this collected information was then synthesized and thematically organized in a special chart. These tasks were performed by the other two authors (APG and AGR) who were also previously calibrated. We did not any kind of quality appraise of included articles. Any discrepancy occurred during these two stages was resolved by discussion and consensus with a third author (MMB).

## Results

**Figure 1:**
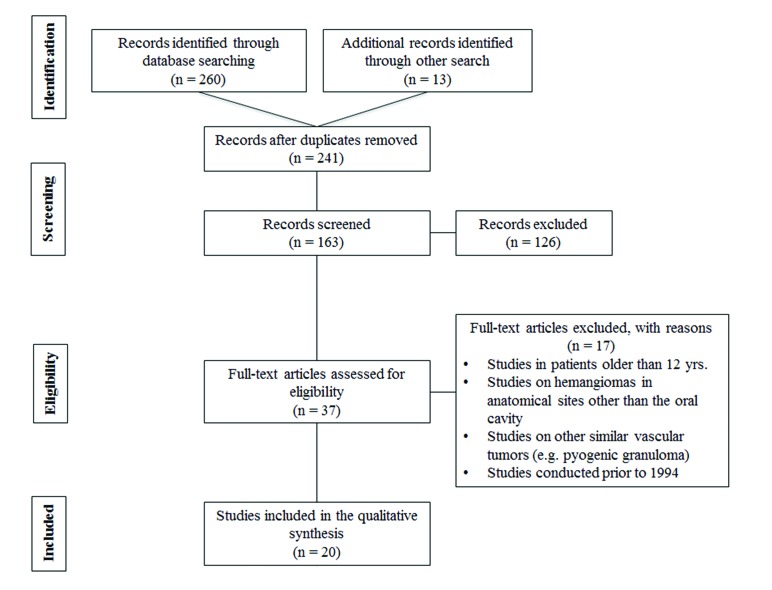
PRISMA flow diagram of literature search.

In this review, the authors initially found 241 references. However, after the processes of duplication removal, screening and exclusion, 37 records were included for data extraction and full-text assessment. Finally, we selected 20 English language references, which met the inclusion criteria and considered as relevant and most representative for the presented clinical topic. Fig. 1 summarizes the screening and selection processes as a PRISMA flowchart. The selected studies were published from 1999 to 2019.

The included articles were from 13 countries, spanning North America, Central and Eastern Asia, Europe, and North Africa. In terms of health science discipline, most studies were identified as pediatric dentistry, oral medicine/pathology, and maxillofacial surgery-oriented; six articles were multidisciplinary. Regarding the methodological design, most studies were retrospective cohorts with chart reviews, case reports, and narrative reviews. No RCCTs were found. General information and characteristics of the included articles are described in Table 1 (4-6,10-16,20-29). After the analysis of the included articles, we identified three fundamental themes associated to the management of pediatric oral hemangiomas: (i) clinical characteristics, differential diagnosis, and histopathological findings; (ii) evolution and complications; and (iii) current available treatment modalities. These issues will be extensively discussed in the next section.

**Table 1:**
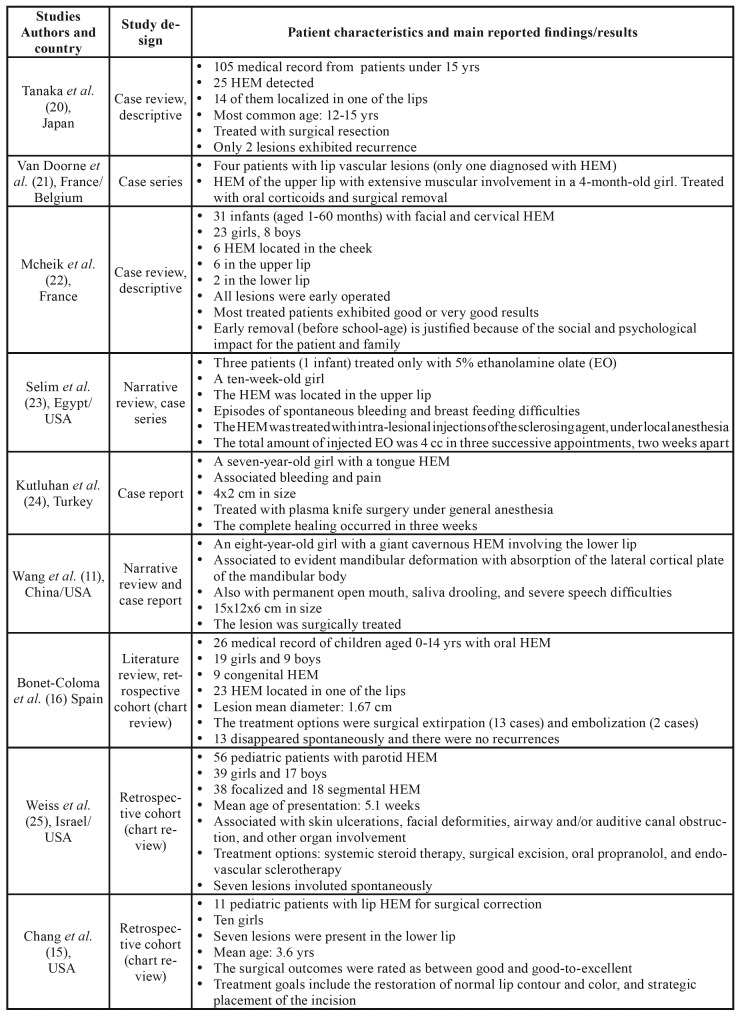
Relevant extracted characteristics and main findings from included studies.

## Discussion

According to the American Academy and Pediatric Dentistry, the management of oral cavity tumor-like conditions in children is determined by diverse anatomical and physiological differences from those of adult patients (1). First, pediatric oral tumors usually grow faster and are less predicTable in behavior; in the second place, although the same physiological factors that affect lesion growth are present, these can play a more favorable role in healing after surgical removal; finally, children are more resilient and heal more quickly than adults.

As above mentioned, the present scoping review determined three principal issues about the pediatric oral hemangiomas to be approached and discussed: (i) clinical characteristics, differential diagnosis, and histopathological findings; (ii) evolution and complications; and (iii) current available treatment modalities.

i. Clinical characteristics, differential diagnosis, and histopathological findings.

Diagnosis of HEM is based on a comprehensive history and clinical examination of the lesion. Only a lesion monitoring or conservative management is suggested for this tumor due to its propensity for spontaneous regression in children (2). Pediatric dentists and maxillofacial surgeons participate, as part of a multidisciplinary health care team, in the diagnosis and treatment of affected children, especially if the tumor endangers the dentition development or when bone tissue is involved (2,10). Therefore, it is mandatory a precise diagnosis –through clinical and imaging assessment– of the type of oral vascular lesion or tumor because it may considerably influence the treatment planning (12). Regardless of the pediatric patient’s age, it is imperative to institute an accurate working diagnosis for each individual oral lesion detected, in order to differentiate between developmental anomalies, reactive or inflammatory lesions, and neoplastic tumors; in this regard, intra-oral soft tissue swellings are frequently overlooked and determined as inflammatory lesions (27). On the other hand, it has been reported that children with neurofibromatosis type 1 (or Von Recklinghausen's disease) are susceptible to a variety of benign tumors, including face and oral skin HEM (30,31).

**Figure 2:**
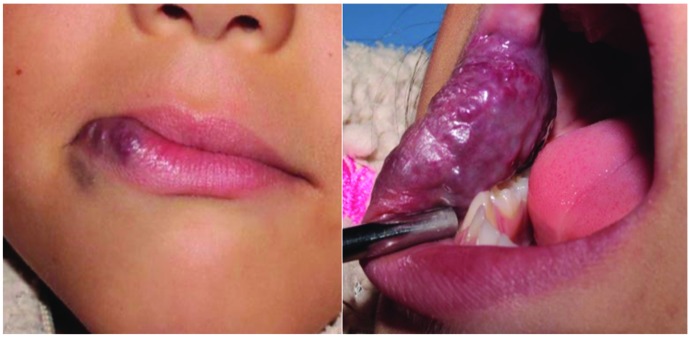
Clinical images of a HEM in the right side of the face, involving the lower lip –crossing the vermilion-skin junction–, and the cheek mucosa. The patient, a 5-year-6-month girl, was referred to our pediatric dentistry clinic (Pediatric Dentistry Postgraduate Program; Faculty of Dentistry; University of San Luis Potosi, Mexico). According to the parents, the lesion was no present at the birth; it was noted around six months earlier and showed rapid growth. On clinical examination, the intraoral bulge felt firm and the overlying skin showed a purple discoloration. The patient and parents did not report any history of ulceration or hemorrhage episodes, nor eating, speaking or respiratory problems. Thus, it was decided to closely observe the lesion for 6 to 9 months, waiting for a spontaneous involution.

HEM may be located superficially or deeply (21,23). Most superficial oral mucosal lesions are manifested as a well-circumscribed, firm, isolated, and raised dark red lesion (macula, papule, or nodule –depending on the congestion degree and deepness into the tissue–) that is rubbery on palpation (2,9); some of these clinical features can be observed in Fig. 2, which corresponds to a female child attending our pediatric dentistry clinic a few months ago. For superficial HEM, particularly those located in the skin of the parotid gland region, diascopy can be employed for diagnostic purposes (32,33).

Vascular lesions tend to blanch in response to the application of a firm pressure by a flat transparent instrument –for example, a glass slide–, during 1 or 2 minutes. When the instrument is removed, the lesion remains pale for a few seconds; then, it slowly starts refilling again from the feeder vessels. According to Tanwar *et al*. (33), diascopy can determine whether the color of a lesion is due to blood present in the vessels (as in HEM) or to extravasated blood present in the tissues: the former case will blanch on pressure and the later will not. However, in problematic identification cases, diagnosis of HEM should be confirmed through imaging methods such as Doppler ultrasound, magnetic resonance imaging, or angiography (13,25).

When HEMs are in the deeper dermis, they are manifested as a slight discoloration of the overlying skin (21). HEM, particularly the capillary type, should be differentiated from other vascular tumors, particularly from the pyogenic granuloma (PG; also called * lobular capillary*). Histopathologically, both lesions resemble each other however PG is usually much smaller and more reactive in nature than HEM, it appears mostly in the gingiva, after the first year of life, and has a typical history of a non-healing/bleeding wound (23,34); furthermore, unlike HEM, PG is predominantly an epithelial rather than an endothelial lesion, and exhibits immunocytochemical and ultrastructural differences in relation to HEM.

Although HEM and arteriovenous malformations (AVM) are considered clinically similar, both lesions exhibit some different histochemical and microscopic characteristics, which can help establish a definitive diagnosis. AVMs are the result of errors in morphogenesis of arteries and venous, while HEMs are a consequence of a derangement in angiogenesis with proliferation of vascular elements. In their anatomic pathology study, Adegboyega and Suimin mentioned that the presence of arteries, arterioles, and intralesional nerves is characteristic of AVM, but not observed in HEM. Additionally, the endothelial lining of HEM is plump, and when cultured, they proliferate and form tubular structures like vascular channels; on the other hand, the endothelial cells lining AVM are quiescent flat cells that neither proliferate nor differentiate into vascular structures (35).

Gliomas and other malignant vascular lesions must be also considered in the differential diagnosis (23). In this same regard, pediatric oral HEMs rarely require imaging for differential diagnosis. However, in some dubious or borderline cases imaging is necessary to assess the extent and penetration of the lesion, the degree of vascularity, as well as its evolution. Ultrasound and associated Doppler spectra are the most employed imaging tools for performing these actions, and also for monitoring the treatment effects on the lesion (6). Regarding to this, ultrasound can show the highly vascular nature of HEM, evidenced by the presence of multiple prominent vessels (36); thus, this diagnostic method is very useful because it can clearly discriminate if a tumor is cystic (rather vascular or lymphatic malformation) or solid (rather HEM) (6).

Histopathologic/microscopic features.

In the present scoping review, seven articles (7,12,26,27,34,37,38) were identified in which a histopathological or microscopic evaluation was performed; all of them were clinical cases on pediatric oral HEM, particularly the capillary type. According to different authors (4,23,37), HEMs may present a spectrum of histopathological findings, consisting in general of hyperplastic endothelial cells exhibiting increased mitotic activity (13). In their simplest classification, HEMs are classified into three types: capillary, cavernous (large-vessel), and mixed, according to the size of vascular spaces. Capillary HEM has numerous small vessels (10-100 microns in diameter) supported in a connective tissue stroma of varying density. Its vessels have walls 1-3 cells thick, and tend to run in parallel; there is also a single layer of endothelial cells with no shedding and no aplasia. Cavernous HEM possesses a similar appearance but the vessel walls are thinner and the lumina are larger; its cavernous vessels or *sinusoids* are lined by epithelial cells, separated by a thin layer of connective tissue septa. In general, the cavernous HEM is larger and more diffuse than the capillary HEM (11). Mixed HEMs contain both components and may be more common than the pure cavernous lesion (37).

Following the AAPD guidelines for HEM cases, the provisional diagnosis is carried out through an in-depth clinical history, assessing the risk factors and documenting the clinical signs and symptoms of the lesion. Then, a list of potential lesions with similar features is rank-ordered from most likely to least likely diagnosis. The condition that is considered to be the most likely one becomes the working diagnosis and determines the initial management approach (1). For difficult cases, immunohistochemical analysis (e.g. expression of human glucose transporter one protein or GLUT-1) and ultrasound imaging are employed (3,10). GLUT-1 is an immunological marker expressed by HEM’s endothelial cells frequently employed in tissue biopsies to differentiate vascular tumors from malformations; a positive stain for GLUT-1 excludes vascular malformations and is suggestive of HEM. This tool is considered as a helpful additional indicator for diagnosing HEM (3). However, this positive result is also possible for other vascular lesions, including epitheloid hemangioendotheliomas, angiosarcomas, and angiokeratomas; so, the final diagnosis for HEM should be made through the interpretation of all clinical and diagnostic features, and not based on GLUT-1 staining alone (6,39).

ii. Evolution and complications.

As previously stated, it has been suggested that HEMs have three different development stages: proliferative, involuting, and involuted. The proliferative phase is characterized by rapid endothelial cell divisions, displaying a tenfold increase in mast cell concentration over the lesions and the normal tissue. During the involuting phase, endothelial cell activity decreases and cellular parenchyma is substituted by fibrofatty tissue (23). Recently, diverse biological positive and negative regulators of angiogenesis have been identified for every HEM biological evolutional phase (e.g. CD31, the von Willebrand factor, the basic fibroblast growth factor or BFGF, the proliferating cell nuclear antigen, and the vascular endothelial growth factor or VEGF); it is believed that the imbalance of these regulators influences the lesion’s pattern of growth (23,25,40).

Oral HEMs may be associated with other craniofacial conditions, such as anterior open bite, salivary leakage, or diverse facial unaccepTable deformations, as well as chewing and speaking disabilities (24). As a consequence, these anomalies can origin strong psychological impacts and inadequate social integration of the affected child. Weiss *et al*. (25) mention that the treatment of parotid hemangiomas is really challenging due to their profuse growth, which may cause significant shunting; in turn, this shunting may result in congestive cardiac failure. According to Püttgen el at. (14), ulcerations are also a common complication, occurring in up to 25% of pediatric patients at referral centers. Other mentioned complication is the auditory canal obstruction in cases of parotid gland HEMs (25). On the other hand, although most HEMs are benign processes, a small group of them represents serious or potential malignant neoplastic conditions (14).

In cases of persisting HEM during the adulthood life, the dentist’s clinical behavior should be focused on the possible close proximity of the lesion to teeth, periodontal bone, and oral mucosa. According to Elias *et al*. (10), dental extractions and incisional biopsies are prone to significant hemorrhages; therefore, the practitioner must be aware and prepared to deal with this adverse reaction or to refer the affected patient to another oral health specialist for more proper management and better prognosis.

iii. Currently available treatment modalities.

A total of nine experimental and observational/longitudinal studies were detected in this scoping review dealing with the treatment (surgical or pharmacological) and follow-up of oral and maxillofacial hemangiomas in pediatric patients (14,15,22,23,25,40-43). Although it has been reported that around 50% of HEMs regress spontaneously with no permanent damages -thus no treatment is required-, it is plenty justified a close observation of all the lesions (22). Surgical and conservative non-surgical treatment options, alone or combined, are reserved only in cases of severe ulcerations or infections, pain, uncontrolled bleeding, airway obstruction, or significant cosmetic deformities (16). For example, non-surgical interventions have the main objective of limiting the cell proliferation, limiting thus the lesion size and its distortive effect, and minimizing the risk of ulceration (15). One of the medical treatments applied nowadays for oral HEMs in children is the corticoid therapy (e.g. systemically or through intralesional injections). Injected corticosteroids are widely used mainly for inducing an early involution for large and rapidly growing HEMs, especially during the first months of life. However, Mcheik *et al*. (22) and Weiss *et al*. (25) do not encourage this therapy because they have frequently observed the appearance of ulceration of the lesion after the corticoid injection. The systemic administration of prednisone or prednisolone has been associated to common and severe side effects, such as weight/height growth retardation, personality alterations, gastric irritation, and opportunistic infections; also it is frequent the lesion rebound growth after cessation of therapy (25). Other non-surgical therapies that have been advocated are the endovascular sclerosing agents, such as 5% ethanolamine oleate, for the relief of HEM symptoms or as a good preparation for further surgery (23,44); the interferon alfa-2a, indicated for the treatment of life-threatening or corticoid-resistant HEM, however irreversible spastic diplegia has been reported with its use; and laser applications, which have been successfully employed for both superficial and deep HEMs (22,45). More recently, bleomycin, beta-adrenergic blockers, polidocanol, and oral propranolol have been introduced, with results so far very promising in terms of therapeutic efficacy and safety in children (25,29,40,42,43). Embolization, cryotherapy, radiations, and chemotherapy (e.g. vincristine) have been also tested (24).

Another treatment strategy of choice for HEMs is surgical excision. Lesions located in the lower lip, upper lip (involving the vermilion-border and sometimes the nose tip), and the parotid/ear region are notoriously slow to disappear and commonly need early surgical removal. Additionally, the early surgical intervention is indicated if the HEM appearance has not changed after two years or when the tumor has not responded to non-surgical therapies. Surgery is also necessary for removing residual fibrofatty tissue and contour defects after the natural involution of HEMs (13,15).

The management of oral hemangiomas does not always fall within the scope of the pediatric dentistry field. Approximately 12% of pediatric oral HEMs are complex and must be referred to other dental specialties for evaluation and management (14). However, many oral medicine/pathology specialists often lack capability and experience to adequately treat young children; thus, it is essential that pediatric dentist practitioners must be part and collaborate, as part of a multidisciplinary dental/medical team, with the purpose of ensuring that affected children are opportunely diagnosed and treated, contributing thus to increase the patient’s quality of life. On the other hand, it has been mentioned that in many towns or cities, particularly of developing countries, specialized clinics combining pediatric dentistry and oral medicine, specific for the management of rare oral mucosal and peri-oral lesions in children, are uncommon or only available in select institutions (46).

In summary, and according with the retrieved information, the administration of pharmacological agents (orally or using endovascular techniques with low doses of propranolol or polidocanol), alone or in combination with conservative surgical treatment, are currently the standard management approach for HEM and other benign vascular lesions in the oral and maxillofacial areas of infants and children; these modalities have demonstrated to be effective and safe in several well-controlled clinical studies with adequate follow-up periods.

Strengths and weaknesses.

To our knowledge, this scoping review represents one of the first attempts to summarize the most current and relevant evidence about the management of intraoral and peri-oral hemangiomas in the pediatric population. In view of the fact that the volume of published information related to this clinical topic is significant, we pretend that the present review’s findings contribute for a future and more critical and systematic analysis of evidence, for the benefit of clinicians and researchers. We chose this research design because of the broad nature of the clinical topic; this type of review is considered suiTable for stating an overview of the current knowledge in a particular health science area, and for providing a concise qualitative analysis.

As many scoping reviews, the authors did not perform any type of critical appraisal to assess the risk of bias of selected articles; thus, some of be included studies might be prone to bias and confounding. In this regard, we often found noticeable differences among studies relative to pediatric hemangiomas in terms of diagnostic criteria and lesion nomenclature. We should recognize that we arbitrarily took methodological some decisions about the organization and analysis processes for the final selection the articles, based sometimes on our own criteria and experience; for example, we considered to include only studies that encompassed children aged less than 12 years. We are aware of the amount of missing potential relevant articles related to the chosen clinical issue approached here; it is possible that our search terms or keywords have filtered out important studies because they did not appear in the title or abstract. Although some of these works could provide useful information, we are confident that the articles included in the present scoping review are valuable enough to synthesize the most important and current knowledge focused on the management of oral hemangiomas in pediatric patients. Finally, this scoping review only included English language studies. As a result, the inherent geographic and cultural diversities of the article sample could be limited. Regarding this, it has been assumed that most dental researchers, who desire to present their results and findings, publish their studies in English since it is the internationally accepted language to report scientific evidence (47).

## Conclusions

As part of the clinical practice, pediatric dentistry practitioners should be familiar with those unusual oral tumor-like vascular entities, such as HEMs. These oral-care specialists should participate in the institution of proper early diagnosis and the design of the management approach during the multidisciplinary clinical decision-making process. The final aim will be to provide optimal therapeutic management and reach adequate psychological, functional and esthetic outcomes for the affected infant or child, without future complications or adverse permanent events.
